# Accurate heterogeneous dose calculation for lung cancer patients without high‐resolution CT densities

**DOI:** 10.1120/jacmp.v10i2.2847

**Published:** 2009-04-30

**Authors:** Anneyuko I. Saito, Jonathan G. Li, Chihray Liu, Kenneth R. Olivier, James F. Dempsey

**Affiliations:** ^1^ Department of Radiation Oncology University of Florida Gainesville FL U.S.A.; ^2^ ViewRay Incorporated Oakwood Village OH U.S.A.

**Keywords:** lung cancer, bulk density, CT, treatment planning, electron density, magnetic resonance imaging

## Abstract

The aim of this study was to investigate the relative accuracy of megavoltage photon‐beam dose calculations employing either five bulk densities or independent voxel densities determined by calibration of the CT Houndsfield number.

Full‐resolution CT and bulk density treatment plans were generated for 70 lung or esophageal cancer tumors (66 cases) using a commercial treatment planning system with an adaptive convolution dose calculation algorithm (Pinnacle^3^, Philips Medicals Systems). Bulk densities were applied to segmented regions. Individual and population average densities were compared to the full‐resolution plan for each case. Monitor units were kept constant and no normalizations were employed. Dose volume histograms (DVH) and dose difference distributions were examined for all cases.

The average densities of the segmented air, lung, fat, soft tissue, and bone for the entire set were found to be 0.14, 0.26, 0.89, 1.02, and $1.12\;g/{\text{cm}}^{3}$, respectively. In all cases, the normal tissue DVH agreed to better than 2% in dose. In 62 of 70 DVHs of the planning target volume (PTV), agreement to better than 3% in dose was observed. Six cases demonstrated emphysema, one with bullous formations and one with a hiatus hernia having a large volume of gas. These required the additional assignment of density to the emphysemic lung and inflammatory changes to the lung, the regions of collapsed lung, the bullous formations, and the hernia gas.

Bulk tissue density dose calculation provides an accurate method of heterogeneous dose calculation. However, patients with advanced emphysema may require high‐resolution CT studies for accurate treatment planning.

PACS number: 87.53.Tf

## I. INTRODUCTION

One of the most influential developments in the evolution of radiotherapy was the introduction of the X‐ray computed tomography (CT) simulator into the radiotherapy treatment planning process, which provided the opportunity to plan treatments based on a 3‐dimentional (3D) model of the patient's anatomy. With the advent of CT‐based treatment planning, it was argued in the early 1980s that determining the electron density of a patient's tissues via CT imaging was necessary for a computational accuracy of better than 5%.^(1)^ Of course, this contention was made before the widespread availability of more advanced and accurate superposition dose computation algorithms,^(2)^ which have been shown to be comparable, if not equivalent, with the implicitly more accurate Monte Carlo simulation when appropriately commissioned.^(3)^ Today, all modern commercially available external beam treatment planning systems provide the ability to calibrate a CT scan for electron density determination. It is well established that accurate dose computation for treatments in the lung require tissue heterogeneity corrections in the dose computation algorithm.^(4)^ ^,^ ^(5)^ However, in this study we will challenge the current paradigm of requiring individually measured electron densities for all of the voxels in a patient determined via a calibrated CT image. Instead, we examine the use of bulk density corrections applied to the different types of tissue present in the patient. This technique has already been demonstrated for MRI‐based prostrate cancer treatment planning.^(6)^ ^,^ ^(7)^ We now extend this approach to treatment planning for lung cancer patients, which clearly relies more heavily on heterogeneity correction for accurate treatment planning.

The aim of this study is to investigate the relative accuracy of megavoltage photon beam dose calculations using bulk densities applied to five distinct regions (air, lung, fat, soft tissue, and bone) compared to dose calculations performed with a full set of individually determined voxel electron densities from a calibrated CT scan. We are investigating the accuracy of this bulk density dose calculation technique for application in an on‐board MRI image‐guided radiation therapy (IGRT) device that is under development at our institution. This IGRT device will utilize real‐time MRI taken during radiation delivery to compute the dose delivered to the patient. Segmented MRI imaging studies can be used to identify bulk density regions of air, lung, fat, soft tissue, and bone in the patient, but the high‐resolution density information of CT imaging is not easily accessible with this technique. Our hypothesis is that bulk densities can provide an accurate method of heterogeneous photon beam dose calculation, even in cancer patients with tumors in the thorax.

## II. MATERIALS AND METHODS

CT planning image studies for 66 lung cancer and esophageal cancer cases demonstrating 70 gross tumor volumes treated at our institution were analyzed anonymously in this study. All patients had undergone helical CT scans (Professional Series P220F Philips Medical Systems, Eindhoven, Netherlands) without contrast enhancement, with an axial‐plane image‐matrix size of 512×512 with an in‐plane pixel size of 1.1 mm and slice thickness of 3.0 mm covering the entire thorax and a superior portion of the abdomen.

Full CT resolution and bulk electron density treatment plans were generated for these 66 patients using a commercial treatment planning system with an adaptive convolution dose‐calculation algorithm (Pinnacle^3^, Philips Medicals Systems, Eindhoven, Netherlands) employing an isotropic 4 mm dose calculation grid. Bulk electron densities were applied to regions identified by an isodensity segmentation tool for each case. In this manuscript, we use the phrase “density” to indicate relative electron density on a scale where the electron density of liquid water is 1.0.

The following procedure was performed for each case. First, the body was contoured as a soft‐tissue structure from 1 slice superior to the lung apex to 1 slice inferior to the lung base. Then, the trachea was manually contoured as an “air density” structure slice‐by‐slice, with special care not to include cartilage. The occasional occurrence of gastrointestinal gas was ignored as it was found that the dosimetric effect of gastrointestinal bubbles was insignificant.^(8)^ Next, the fat and bone were contoured using an auto‐contouring tool with thresholds of 600–800 and 1080–4500, respectively (Note: In Pinnacle, to avoid using negative numbers, 1000 is added to all CT numbers. Thus, water density, which is usually 0HU in diagnostic CTs, is 1000). Finally, the soft tissue bulk density structure was produced by subtracting the air, lung, fat, and bone bulk density regions from the body contour. For each patient, the mean bulk density was calculated (the automatic calculated data from Pinnacle^3^ was used) and recorded as the individual average density. Using these data, the average density of each bulk density of the whole set of 66 patients was calculated as the population average.

**Figure 1:**
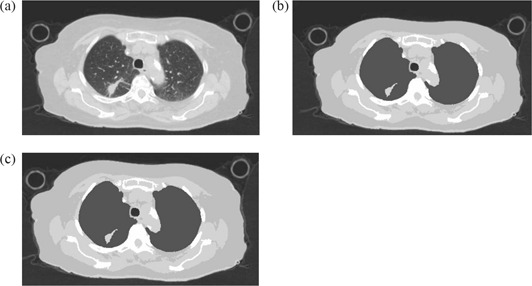
Examples of images used for creating the three plans. an original full‐resolution CT image (lung window); (b) a CT image where the electron density of each 5 bulk density was forced to be the same as each patient's average electron density of each bulk density area (IndAv.); (c) a CT image where the electron densities of each 5 bulk density was forced to be same as the average electron density of each bulk density area for the entire population (PopAv.).

For each case, 3 plans were created: 1) original full‐resolution CT plan, referred to as the original plan (Org.) (Fig. 1(a) and defined as a plan created based on the full CT resolution electron density information; 2) individual average plan (IndAv.) (Fig. 1(b), which was a plan created using the five bulk densities where the electron densities of each area were forced to be the same as each patient's individual average; and 3) population average plan (PopAv.) (Fig. 1(c), which was a plan created using the five bulk densities where the electron densities of each area were forced to be the same as the population average. Monitor units in the planning system were kept constant to Org., and no normalizations or adjustments were employed.

**Figure 2:**
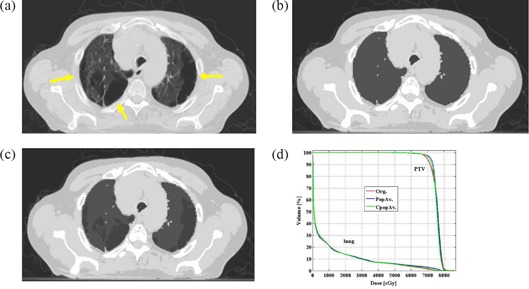
Example of a patient with bullous formations (yellow arrows) in both lungs. original treatment planning CT image (lung window); (b) treatment planning CT image of the same slice as with the 5 bulk densities applied; (c) treatment planning CT image of the same slice as and where air density is applied to the bullous formations of both lungs; (d) DVH comparing the PTV and lung of the 3 plans.

**Figure 3:**
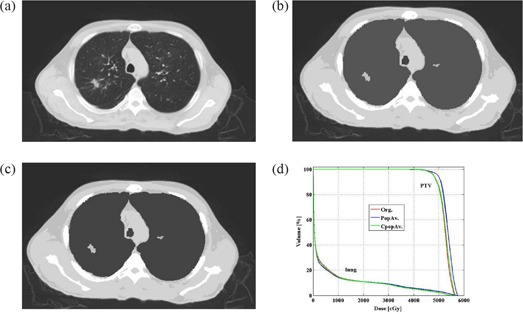
An example of a patient with emphysema. Fig.3(a) original treatment planning CT image (lung window); (b) treatment planning CT image of the same slice as with the 5 bulk densities applied; (c) treatment planning CT image of the same slice as Figs. 3(a) and (b) where the individual average density is applied to the lung; (d) DVH comparing the PTV and lung of the 3 plans.

**Figure 4:**
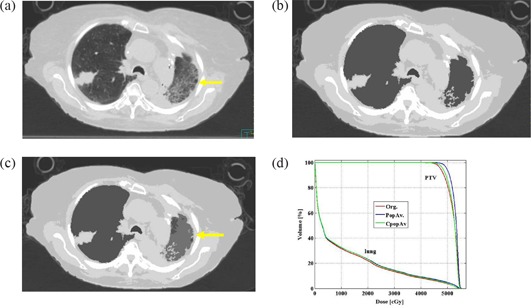
An example of a patient with pneumonia (yellow arrow) in the left lung. original treatment planning CT image (lung window); (b) treatment planning CT image of the same slice as 4a with the 5 bulk densities applied; (c) treatment planning CT image of the same slice as Figs. 4(a) and (b) where the individual average density is applied to the lung and $0.43\;g/{\text{cm}}^{3}$ is applied to the ground glass appearance area (yellow arrow); (d) DVH comparing the PTV and lung of the 3 plans.

**Figure 5:**
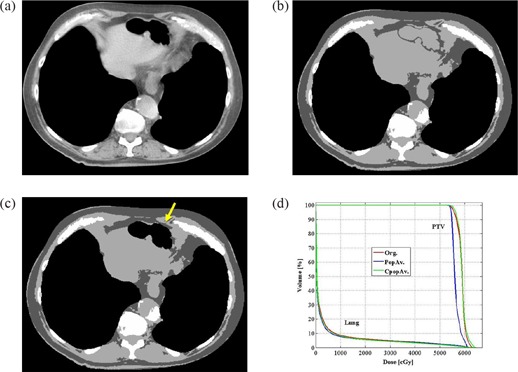
An example of a patient with a hiatus hernia filled with a large amount of gas. original treatment planning CT image (mediastinum window); (b) treatment planning CT image of the same slice as 5a with the 5 bulk densities applied; (c) treatment planning CT image of the same slice as 5a. and b. where the air density is applied to hiatus hernia (yellow arrow); (d) DVH comparing the PTV and lung of the 3 plans.

In cases presenting with lung or gastrointestinal pathologic conditions, such as bullous formation (7 patients), pneumothorax (2 patients), or emphysema (12 patients, 2 of whom also had inflammatory changes of the lung, 1 also had pneumothorax, and 1 also had hiatus hernia), additional corrections were applied. These plans were referred to as corrected modified population average plans (CpopAv.). The correction done for the patient with bullous formations or pneumothorax was to assign air density to the bullous formations or pneumothorax (Figs. 2(a) – 2(d)). The correction done for the emphysema patients, who had obvious findings on the planning CT, was to assign the individual lung density to the lung bulk density instead of using the population average density (Figs. 3(a)) – 3(d)). The correction for the patients who had both emphysema and inflammatory changes in the lung was to assign a new density to the inflammatory change area ($0.43\;g/{\text{cm}}^{3}$, Figs. 4(a) – 4(d) and assign the individual average data for the rest of the lung. (The number $0.43\;g/{\text{cm}}^{3}$ was the population average data of all the lung inflammatory changes density of this study.) The correction for the patient with hiatus hernia was done by assigning air density to the gastrointestinal gas (Figs. 5(a) – 5(d)).

A dose volume histogram (DVH) comparison and slice‐by‐slice comparison of the dosimetry of the treatment area were performed for all plans. We specifically investigated the following parameters: the dose covering 95% (D95) or more of the PTV; the mean PTV dose; the percentage of the total lung volume covered with 20 Gy or higher (V20Gy %); and the mean total lung dose. If the dose or volume differences were within 3% of the original plan, the plan was considered an acceptable plan. For the slice‐by‐slice comparison, we calculated the percentage of dose‐grid voxels, which showed more than 5% disagreement in the area with a gradient less than 3% of the maximum dose per mm.

To investigate which attributes of a given case led to a larger observed error in five bulk density dose calculations, we statistically analyzed correlations between the absolute errors of the plans and patient conditions and the absolute errors between Org. and PopAv. The patient condition parameters used were: size of the PTV, size of the lung, disease (lung cancer or esophageal cancer), number of beams, tumor location (fully surrounded by the lung or not), with or without emphysema, with or without bullous formations, with or without inflammatory changes in the lung, individual average electron density of the lung, individual average electron density of the bone, individual electron density of the soft tissue, individual electron density of the trachea, and individual electron density of the fat. For each parameter, the data were divided in two groups: one group which had the same or a lower score than the median of the whole data, and one which had a higher score.

All statistical analyses were computed using Statistical Analysis Systems (SAS) software (SAS Institute, Cary, NC). The correlation between the absolute errors of the plans and each parameter was examined with the Wilcoxon signed‐rank sum test. A p‐value of <0.05 was interpreted as evidence that the observed difference was statistically significant.

## III. RESULTS

**Figure 6:**
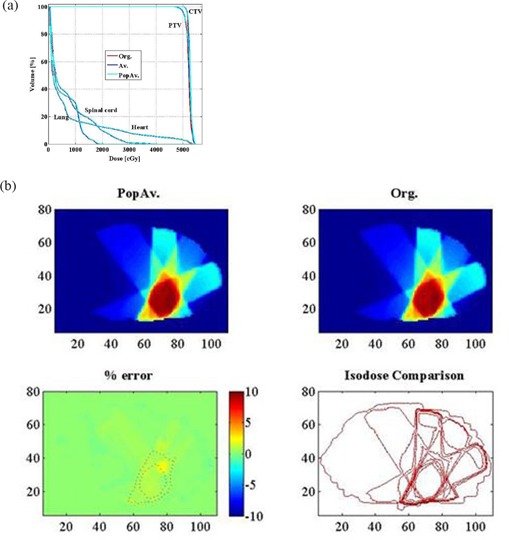
Typical DVH and slice‐by‐slice comparison images. DVH; (b) slice‐by‐slice comparison at the middle slice of the PTV.

Table 1 shows the patients' demographics. Table 2 shows the population average of each bulk density as follows (the number in the parentheses represents the HU number of each density): bone, $1.12\;g/{\text{cm}}^{3}$ (1200); soft tissue, $1.02\;g/{\text{cm}}^{3}$ (200); fat, $0.89\;g/{\text{cm}}^{3}$ (–110); lung, $0.26\;g/{\text{cm}}^{3}$ (–740); and air, $0.14\;g/{\text{cm}}^{3}$ (–860). Tables 3 and 4 summarize the average and percentage differences in dose volume information between the Org. and IndAv. or Popav. plans. In general, only slight differences were observed between the plans with essentially no significant changes for critical structure doses in any case. For the evaluated structures (which included the lung, heart, and spinal cord), no difference larger than 2% in both dose and volume was observed in the DVH for all patients. Figure 6(a) shows a DVH of a typical case with good agreement between plans (the DVH of three plans are displayed as an overlay). However, some significant differences were observed for PTV coverage. In 8 out of 70 targets, greater than 3% differences in PTV doses were observed for the PopAv. comparison: 7 targets (10% of the targets studied) showed larger than 3% difference (maximum difference: 9.70%) in D95 and, if comparing the mean dose, 3 targets (4% of the targets studied) had a difference larger than 3% (maximum difference: 7.34%). Note that one of these had less than 3% in D95, making a total of eight patients with discrepancies greater than 3%. Using individual densities reduced the number of tumors with greater than 3% differences in PTV doses to 6 targets for the IndAv. comparison: 5 targets (7% of the targets studied) showed larger than 3% difference (maximum difference: 6.83%) in D95 and, if comparing the mean dose, 3 targets (4% of the targets studied) had a difference larger than 3% (maximum difference: 5.80%). Note that one of these had less than 3% in D95, making a total of six patients with discrepancies greater than 3%.

**Table 1:** Patient characteristics (N=66).

*Gender*	
Male	46 patients
Female	20 patients
*Race*	
Black	5 patients
White	61 patients
*Age*	
Range	17–88 years
*Body mass index*	
Median	$25\;\text{kg}/m^{2}$
Range	$13–53\;\text{kg}/m^{2}$
*Disease (70 tumors)*	
Esophageal cancer	14 patients
Lung cancer	56 patients
*Lung tumor position*	
Left lobe	25 patients
Mediastium and/or hilum	8 patients
Right lobe	23 patients
Upper lung field	35 patients
Middle lung field	17 patients
Lower lung field	4 patients
*Esophageal position*	
Middle esophagus	2 patients
Inferior esophagus	12 patients
*Existing lung and gastrointestinal tract condition in treatment area (some cases had 2 or more simultaneous conditions)*	
Bullous formations	7 patients
Emphysema^a^	12 patients
Inflammatory changes (ground glass appearance)	15 patients
Pneumothorax	3 patients
Hiatus hernia	1 patients
Pulmonary effusion	4 patients
*Total dose*	
Median	4500 Gy
Range	2000–7440 Gy
*Energy*	
Only 6 MV	50 patients
Mixture with higher energy	16 patients
*Number of beams*	
Median	4
Range	2–9

a Patients with evident findings on CT.

**Table 2:** Electron density and volume data of the 5 bulk density categories (n=66).

	*Electron density (g/cm^3)^*		*Volume (cm^3)^*	
	*Mean*	*SD*	*Mean*	*SD*
Bone	1.12	0.02	1133.51	289.57
Soft tissue	1.02	0.01	6351.64	1548.15
Fat	0.89	0.01	3960.47	2225.46
Lung	0.26	0.06	3368.99	1351.63
Air (trachea)	0.14	0.04	26.56	11.09

SD=standard deviation

**Table 3:** Dosimetric comparison between original full‐resolution CT plans (Org.), 5 bulk density plans using each patient's average data (IndAv.), and 5 bulk density plans using population average data (PopAv.).

	*Org*.		*Av*.		*PopAv*.	
	*Mean*	*SD*	*Mean*	*SD*	*Mean*	*SD*
PTV D95^a^ (cGy)	4036	1403	4081	1430	4082	1443
PTV mean dose (cGy)	4290	1388	4322	1399	4315	1402
Lung V20Gy%^b^ (%)	13.70	8.75	13.53	8.76	13.84	8.67
Lung mean dose (cGy)	784	433	780	436	791	437

a PTV D95; Dose covering 95% or more of the PTV.

b Lung V20Gy%: Percentage of the lung volume covered with 20Gy or more.

PTV=planning target volume;SD=standard deviation

**Table 4:** Absolute difference between original full‐resolution CT plans (Org.), 5 bulk density plans using each patient's average data (IndAv.), and 5 bulk density plans using population average data (PopAv.).

	*Org. vs. IndAv*.			*Org. vs. PopAv*.		
	*Mean*	*SD*	*Nr*.^a^	*Mean*	*SD*	*Nr*.^a^
PTV D95^b^ (%)	1.18	1.24	5	1.2	1.7	7
PTV mean dose (%)	0.89	0.89	3	0.87	1.09	3
Lung V20Gy%^c^	0.49	0.49	0	0.23	0.34	0
Lung mean dose (%)	0.63	0.57	0	0.23	0.41	0

a Number of tumors where the difference was 3% or larger.

b PTV D95; Dose covering 95% or more of the PTV.

c Lung V20Gy%: Percentage of the lung volume covered with 20Gy or more.

PTV=planning target volume;SD=standard deviation

All eight patients with PTV dosimetry exhibiting more than 3% dose difference for the PopAv. comparison presented with some lung or gastrointestinal tract conditions, such as bullous formation (1 patient), hiatus hernia with emphysema (1 patient), emphysema alone (3 patients), inflammatory changes of the lung with emphysema (2 patients), and inflammatory changes of the lung with emphysema and pneumothorax (1 patient). The largest difference between PopAv. and Org. was observed in a patient who had both inflammatory change and emphysema (D95, 10% difference; mean PTV dose, 7% difference). When these corrections were applied to the pathologic structures in these patients (Figs. 2–5), as shown in Table 5, no patient showed a difference of 3% or larger in the D95 and mean dose of PTV.

**Table 5:** Absolute difference between original full‐resolution CT plans (Org.) and two types of 5 bulk density plans in patients with special conditions: PopAv., where population average data were used, and CpopAv., where a condition‐oriented special modification was applied.

*Condition*	*No. of patients*	*Parameter*	*Org. vs. PopAv*.			*Org. vs. CpopAv*.		
			*Mean*	*SD*	*Nr*.^a^	*Mean*	*SD*	*Nr*.^a^
Emphysema alone	8	PTV D95^b^ (%)	2.26	2.70	2	1.04	1.13	0
		PTV mean dose (%)	1.27	1.42	1	0.41	0.49	0
Emphysema+Inflammatory changes	2	PTV D95^b^ (%)	7.86	2.63	2	1.85	0.57	0
		PTV mean dose (%)	5.13	3.12	1	2.13	0.58	0
Emphysema+hiatus hernia	1	PTV D95^b^ (%)	0.81	‐	0	0.35	‐	0
		PTV mean dose (%)	3.21	‐	1	0.96	‐	0
Bullous formations/pneumothorax^c^	10	PTV D95^b^ (%)	1.49	1.56	2	1.13	0.99	0
		PTV mean dose (%)	0.81	0.74	0	0.75	0.72	0

a Number of patients where the difference was 3% or larger.

b PTV D95; Dose covering 95% or more of the PTV.

c The one patient who has both emphysema and pneumothorax is included here.

PTV=planning target volume;SD=standard deviation

When a slice‐by‐slice comparison of the dosimetry of the treatment area was performed for the IndAv. comparison, there was only 1 case where the percentage of voxels with dose disagreement of more than 5% was larger than 1% (mean, 0.2%; SD, 0.32%). For the PopAv. comparison, there were 4 cases where the disagreement area of more than 5% was larger than 1% (mean, 0.33%; SD, 0.68%). Figure 6(b) shows a slice‐by‐slice comparison between Org. and PopAv. for a typical case (the middle slice of the target is demonstrated). When employing CpopAv. for cases with pathological conditions, the comparison resulted in 1 case where the percentage of voxels with disagreement was larger than 1% (mean:0.18%, SD:0.26%).

The parameters, which show statistically significant differences in the PopAv. comparison, are summarized in Table 6.

**Table 6:** Patients' conditions that could lead to a larger risk of dose calculation error.

*Condition*	*No. of patients with this condition*	*p‐value*	
Lung volume larger than 3396cc	32	PTV D95^a^ (%)	p=0.01
		PTV mean dose (%)	p=0.04
Pathological condition of emphysema	12	PTV D95^a^ (%)	p<0.01
		PTV mean dose (%)	p<0.01
Electron density of the lung $0.25\;g/{\text{cm}}^{3}$ or lower	31	PTV D95^a^ (%) PTV mean dose (%)	p<0.01 p=0.03
Fully surrounded by the lung	22	PTV D95^a^ (%)	p=0.04
		PTV mean dose (%)	p=0.54

a PTV D95 ‐ dose covering 95% or more of the PTV.

## IV. DISCUSSION

In comparing the plans created with the five bulk densities and the Org., there were no patients for whom 2% or more difference was seen in either dose or volume on the DVH of the lung, heart, and spinal cord. However, when investigating the dose to the PTV, 8 cases showed more than 3% difference between the plans. For D95, 93% of the PTVs in IndAv. and 90% of the PTVs in PopAv. had less than a 3% difference compared to Org. For the mean dose of the PTV, it was 96% and 94%, respectively.

All the tumors for which 3% or more difference was observed between Org. and PopAv. had either a pathologic lung condition (eg. bullous formation, pneumothorax, emphysema, an inflammatory change in the lung), or a gastrointestinal change such as hiatus hernia with a large amount of gas. The largest disagreement, seen in a patient with emphysema who also had an inflammatory change in the lung, was 10% for D95 of the PTV and 7% for the mean dose of the PTV. Specific density corrections applied to the pathologic structures were able to resolve all of these problems. In the slice‐by‐slice comparison between Org. and PopAv., four cases showed more than 1% of the voxels with more than 5% difference in a low‐gradient region. The largest difference was observed in a case of emphysema, which was 4.09% of the voxels with more than 5% disagreement in a low‐gradient dose region. If the specific corrections were applied, no patient had more than 2% of voxels with disagreement larger than 5% and, even the worst case, the case with emphysema, showed only 1.37% of the voxels with disagreement larger than 5%.

Correcting the emphysema patients for the CpopAv. plan required the use of the average electron density of each patient's lung from a bulk density determined from the CT high‐ resolution data. Corrections in the other cases could be determined using only MRI information.^(9)^ Consequently, 58 out of 70 tumors (83%), which were in the patients without emphysema, were accurately simulated with only the use of an MRI simulator. For the remaining 12 patients with emphysema, accurate dose computation on MRI data could be achieved as long as the bulk density of the pathologic regions was provided by measurement with an additional CT scan. Out of these 12 patients, only 6 required CT determined bulk densities; however, we could not distinguish between these patients using the MRI data alone.

The univariate analysis of the data showed that the parameters which demonstrated correlation with large discrepancy between Org. and PopAv. were: large lung volume; pathological condition of emphysema; lower electron density of the lung; and lung tumors located such that they were fully surrounded by lung tissue. Emphysema patients have larger lung and lower lung density. Therefore, the first three parameters were obviously correlated. It is also understandable that lung tumors fully surrounded by lung tissue required additional density correction and showed a higher probability of disagreement.

In a previous study by Lee et al.^(6)^ on the accuracy of employing MRI data to compute dose for prostate cancer patients, it was concluded that, if both bone and water were assigned as bulk densities, the difference was less than 2% in all plans; but if only water density was used, the disagreement was larger. The body bulk density area was assigned as 0 HU $\left(1\;g/{\text{cm}}^{3}\right)$, a little lower than in our study $\left(1.02\;g/{\text{cm}}^{3}\right)$. This number was not from the average density of the patients' data; Lee et al. simply assigned water density as done for a non‐heterogeneous correction plan. They also did not find any problems with not making another assignment for fat density even though, according to our findings, this should have been an important factor for a larger population which would include obese patients. Their study included only 5 patients. They may not have included overweight patients in their study, but it is not possible to know this from their published data. We used five bulk densities in this study because, in our previous study with 17 lung cancer patients' CT images where we used four bulk densities (air, lung, soft tissue and bone),^(10)^ patients with high BMIs ($25\text{kg}/m^{2}$ or higher) showed larger errors. As fat is easy to define on MRI, we assume that the addition of the fat density would not cause a large problem in the MRI dose calculation. Another interesting difference was noted between our study and the previous study. The CT number Lee et al. found for bone was 320 HU ($1.32\;g/{\text{cm}}^{3}$ in electron density), which came from the average bone density of the five patients. In our study, the bone density was also the average data of the 66 patients, and was $1.12\;g/{\text{cm}}^{3}$. This difference is most likely due to the difference between the thinner cortical bones in the thoracic area compared to the thicker cortical bones in the pelvic region.

Knowing that population averages of electron densities can produce accurate dose computation is not only valuable to the use of an MRI simulator, but can also provide a way to check the calibration of HU‐to‐density calibrations for CT‐based treatment planning. Of course, heterogeneous plans can be more accurate for patients with certain pathologic conditions, as we have shown in this study, and may require CT data in some cases.^(^ ^11^ ^–^ ^13^ ^)^ However, by employing an accurate method using population base values, the use of the CT simulation could be omitted. CT‐simulator dose calculation is based on the CT number to Hounsfield Unit conversion, which is not only machine and vendor dependent, but also dependent on the CT calibration, which has a potential for additional error. If this step could be omitted, the treatment planning paradigm would be safer.

Our study shows that even for patient with tumors in the thoracic region, treatment planning using only MRI could be accurately performed. The exception is the severe emphysema patients who would still need CTs to obtain the electron density information of the diseased lung in order to assign a density to their lung bulk density area.

## V. CONCLUSIONS

Heterogeneous correction for five bulk densities is an accurate method of radiotherapy treatment planning and can be used in place of full‐resolution CT treatment planning. Additional bulk electron density information may be required for patients presenting with pathologic conditions like emphysema.
